# Employees and the National Insurance Act

**Published:** 1912-03-09

**Authors:** 


### EMPLOYEES AND THE NATIONAL INSURANCE ACT.

### I.—Some Preliminary Points of Interest.

Within little more than four months, namely on July 15 next, unless the date be deferred by his Majesty in Council, the National Insurance Act, 1911, will come into operation, and those who will necessarily be affected by its provisions should immediately take steps to ensure the receipt of the full benefits obtainable under the Act. It is desirable to set out the classes of people affected.

1. Insured Persons.—All persons of both sexes, between the ages of 16 and 65, who are employed within the meaning of the first schedule of the Act must be insured. Employment as defined by the first schedule extends to artisans, mechanics, clerks, nurses, servants, and so forth, who are under contract of service, express or implied, and no matter how they may be paid, whether by the hour, day, week, month, or quarter, except those whose remuneration is at the rate of over £160 per annum and derived from other than manual labour. Such persons are referred to as "employed contributors" in distinction from "voluntary contributors," the latter being those who are entitled to become insured persons by reason of the fact that they are engaged in some regular occupation and are wholly or mainly dependent for their livelihood on the earnings derived by them from that occupation, and have an income from all sources of not more than £160 per annum. The term "voluntary contributor" also includes those who, having been insured for a period of at least five years, are entitled to continue their contributions though their income may exceed £160 per annum.

2. Employers.—The responsibility of seeing that the contributions are paid will rest upon the employer, who has himself to pay a sum of 3d. per week for each employee, the contribution of the latter being 4d. per week in the case of males and 3d. per week for females, with minor exceptions.

*The administration of the benefits* is to be carried out by "Approved Societies" and "Insurance Committees," and the Act has been drafted to secure the greatest benefit to members of approved societies. Compulsory contributors who fail to join an approved society must become deposit contributors.

The advantage of becoming a member of an approved society is best explained negatively—that is to say, by describing the disadvantages of deposit insurance.

All contributions in respect of deposit contributors are the same as for those who join approved societies. But the benefits are greatly modified.

In the first place the deposit contributor must contribute for 26 weeks before he can obtain any benefit. His account is then charged in advance with the sum required by the Insurance Commissioners to provide his share, and that of the employer, of the expenses of administration and the cost of medical attendance and sanatorium benefit for the ensuing year. He will then be entitled to draw on the balance to the credit of his account for sickness, disablement, and maternity benefits until such credit is exhausted. When such credit is exhausted his benefit comes to an end, except for medical and sanatorium benefit, which will remain in force until the end of the then current year, and for so much longer as the Insurance Committee may determine. The extension of the benefit beyond the then current year depends upon the Committee having funds available. Thus the principle of insurance so far as sickness, disablement, and maternity benefits are concerned is non-existent.

Again, on the death of a deposit contributor his or her estate will only benefit by the proportion of the balance standing to the credit of the account attributable to his or her own contributions—that is to say, no account is to be taken of the fund accumulated from the contributions of the employer on his or her behalf, such sum being forfeited. A similar provision is made in the case of deposit contributors permanently ceasing to reside in the United Kingdom. More than this, the special feature of the Act is that all persons joining approved societies below age 50 will be entitled to the same benefit for the same level contribution irrespective of age. Seeing, however, that sickness increases with age, an initial reserve increasing with the age would be required on account of all lives entering upon insurance at ages over 16. These reserves in the aggregate are estimated to amount to £66,642,900 for the United Kingdom, and provision has been made in the Act to liquidate this initial deficiency during the next 18 years or thereabouts. This provision is of special advantage to the older lives and has no counterpart in the deposit insurance section.

In view of these considerations the organisation of approved societies is a matter of pressing importance, and it should receive the attention of the Board of Governors of all voluntary hospitals without delay. Last week we commented on the memorandum circulated by Mr. Harry Johnson, the House Governor and Secretary of the Leicester Infirmary. It was indicated that the Royal National Pension Fund for Nurses had already taken steps for the approval of such a society, to be known as the Nurses' National Insurance Society. This society is intended to apply solely to trained female nurses, and in consequence the large body of those engaged in hospitals and those casually employed are untouched by the scheme in question. The resolution of the Board of the Leicester Infirmary suggesting the formation of an approved society for the combined English hospitals was considered at a meeting of the British Hospitals Association on March 1. It was decided to obtain counsel's opinion on the subject before taking further action. A reference to Clause 28 of the National Insurance Act, section (1) proviso (2) will show at once the prudence and necessity for this step.

Specimen sets of rules for approved societies have been issued by the Insurance Commissioners during the past week for the guidance of those contemplating the formation of societies. We propose to refer to these next week and particularly to the benefits obtainable by trained female nurses.

